# Role of ^99m^Tc-ECD SPECT in the Management of Children with Craniosynostosis

**DOI:** 10.1155/2014/172646

**Published:** 2014-04-16

**Authors:** Mayadhar Barik, Minu Bajpai, Rashmi Ranajn Das, Arun Malhotra, Shasanka Shekhar Panda, Manas Kumar Sahoo, Sadanand Dwivedi

**Affiliations:** ^1^Department of Pediatric Surgery, All India Institute of Medical Sciences, New Delhi 110029, India; ^2^Department of Pediatrics, All India Institute of Medical Sciences, Bhubaneswar 751019, India; ^3^Department of Nuclear Medicine, All India Institute of Medical Sciences, New Delhi 110029, India; ^4^Department of Biostatistics, All India Institute of Medical Sciences, New Delhi 110029, India

## Abstract

*Purpose of the Report*. There is a paucity of data on correlation of various imaging modalities with clinical findings in craniosynostosis. Moreover, no study has specifically reported the role of ^99m^Tc-ECD SPECT in a large number of subjects with craniosynostosis. *Materials and Methods*. We prospectively analyzed a cohort of 85 patients with craniosynostosis from year 2007 to 2012. All patients underwent evaluation with ^99m^Tc-ECD SPECT and the results were correlated with radiological and surgical findings. *Results*. ^99m^Tc-ECD SPECT revealed regional perfusion abnormalities in the cerebral hemisphere corresponding to the fused sutures preoperatively that disappeared postoperatively in all the cases. Corresponding to this, the mean mental performance quotient (MPQ) increased significantly (*P* < 0.05) postoperatively only in those children with absent perfusion defect postoperatively. *Conclusions*. Our study suggests that early surgery and release of craniosynostosis in patients with preoperative perfusion defects (absent on ^99m^Tc-ECD SPECT study) are beneficial, as theylead to improved MPQ after surgery.

## 1. Introduction

Craniosynostosis is the premature closure of one or more of the calvarial sutures with a prevalence of 3 to 6 per 10,000 live births [1, 2]. It can be primary, where a developmental defect during embryogenesis is the cause, or secondary, where the causes can be mechanical including compression of the fetal skull against maternal pelvis; metabolic like rickets, hypophosphatasia, hypercalcemia, anemias, and hyperthyroidism; decreased intracranial pressure as a result of brain atrophy or after shunting procedure for hydrocephalus; and teratogens [1, 2]. Proteins encoded by genes like MSX2, FGFR1-3, TWIST1, and EFNB1 control the intramembranous ossification of the skull, and mutation of these genes may lead to premature sutural and resultant craniosynostosis [3]. About 85% cases are nonsyndromic, occurring as isolated involvement of sutures without any associated extracranial anomaly, whereas remaining 15% occur as syndromic craniosynostosis [4].

Different imaging modalities have been used in the assessment of children with craniosynostosis [5]. Plain X-ray of skull is useful in diagnosis except during the first 3 months of life (due to low mineralization) and the features include perisutural sclerosis, absence of suture, localized breaking, and bony bridging. Computed tomography (CT) scan is a useful modality as it helps in assessing the brain (e.g., parenchyma problems, hydrocephalus, and congenital malformation) and the skull including the sutures simultaneously. MRI is helpful in the assessment of syndromic craniosynostosis as it delineates the soft-tissue structures including brain parenchyma more clearly. Though these imaging modalities are helpful in preoperative structural assessment of the affected subjects, they however are not much of help for assessment of perfusion abnormalities and functional problems (e.g., cognitive, visual) related to the craniosynostosis [6, 7]. Recognition of the latter problems improves surgical outcome and reduces treatment delay [8].

Single photon emission computed tomography (SPECT) using a number of neutral lipophilic radiotracers like ^99m^Technetium-ethylene-cysteine-dimer(^99m^Tc-ECD) and ^99m^Technetium-hexamethylpropyleneamine-oxime(^99m^Tc-HMPAO) has been shown to be very useful for the study of regional cerebral perfusion in a variety of disorders affecting the central nervous system [6, 9]. We have previously shown cerebral hypovascularity in craniosynostosis using ^99m^Tc-HMPAO in seven children [10]. In the present study, we analyzed a large number of children with craniosynostosis by using ^99m^Tc-ECD SPECT and compared it with other imaging modalities as well as clinical parameters.

## 2. Materials and Methods

This was a prospective study conducted on 85 children attending to outpatient department of pediatric surgical unit and clinically diagnosed with craniosynostosis between February 2007 and April 2012 (Table 1). All the affected children had undergone a detailed clinical evaluation including assessment of vision, fundoscopy, and plain X-rays of the skull obtained in four views (AP, lateral, basal, and Towne's) followed by noncontrast computed tomography (NCCT) scan (SOMATOM, Siemens Healthcare, USA) of the head to evaluate the extent of sutural involvement, associated ventriculomegaly, and parenchymal changes. MRI of the brain (MAGNETOM Avanto, Siemens Healthcare, USA) was carried out only in children with syndromic craniosynostosis. Subsequently, ^99m^Tc-ECD Brain SPECT (Symbia T6, Siemens Healthcare, USA) studies were carried out preoperatively in all the children. Psychological assessment was done using the Vineland Social Maturity Scale (VSMS), to assess the social maturity of the child and to assess self-help skills. The AIIMS developmental schedule was prepared to assess the mental performance in terms of quotient (MPQ) of children from 0 to 5 years of age and to calculate the motor, adaptive, language, and personal social quotient separately.

For the ^99m^Tc-ECD Brain SPECT study, each patient received an oral sedation with chloral hydrate (pedicloryl) 50 mg/kg body weight (maximum up to 100 mg/kg) and an intravenous cannula was inserted about 10 min before the isotope injection. The children were kept in semidark, quit room and intravenously administered 350 MBq–550 MBq of ^99m^Tc-ECD. SPECT study was performed between 15 and 30 min of intravenous administration of ^99m^Tc-ECD on an Elscint SP-4 orbit SPECT system with a truncated single head. SPECT images at 360° were acquired with 4° intervals in the step and shoot mode, in a circular orbit. The raw data was normalized for uniformity, center of rotation, and gantry motion correction. Transaxial slices of 10 mm thickness are generated with convoluted back projection reconstruction, using a ramp and hamming filter. A factor of 0.125 was applied for attenuation correction. Single pixel slices in the transaxial, coronal, and sagittal planes were obtained. Length of acquisition varied from 20 to 30 min. All the slices were viewed on a color monitor by three observers [all having master's degree (MD) in nuclear medicine and having experience in handling the SPECT images], and the final interpretation was based on a consensus. An abnormal study included asymmetry on two sides greater than 10% (qualitatively), defect size of more than one slice (1 pixel) in thickness, and extent of the lesion in more than one plane. Institutional ethics committee approval was obtained for the study.

All the children underwent surgery. The surgical procedure involved a linear craniectomy of coronal and metopic suture and parasagittal craniectomy extending to bilateral lambdoid craniotomies when indicated. The frontoorbital segment was detached from the calvarium by dissection at the anterior cranial fossa base, nibbling of the frontosphenoid sutures, and division of the frontonasal and frontozygomatic sutures. This detached segment was advanced 1-2 cm according to the requirements of the individual case by the modified tongue in groove technique. Again, ^99m^Tc-ECD Brain SPECT studies were carried out postoperatively in all the children. All children had uneventful postoperative recovery and were well at follow-up (ranging from 3 months to 15 years). All the descriptive data were entered into Excel sheet. Paired* t*-test was used to compare the MPQ scores before and after surgery. A* P* value of <0.05 was considered significant.

## 3. Results

**Figure 1:**
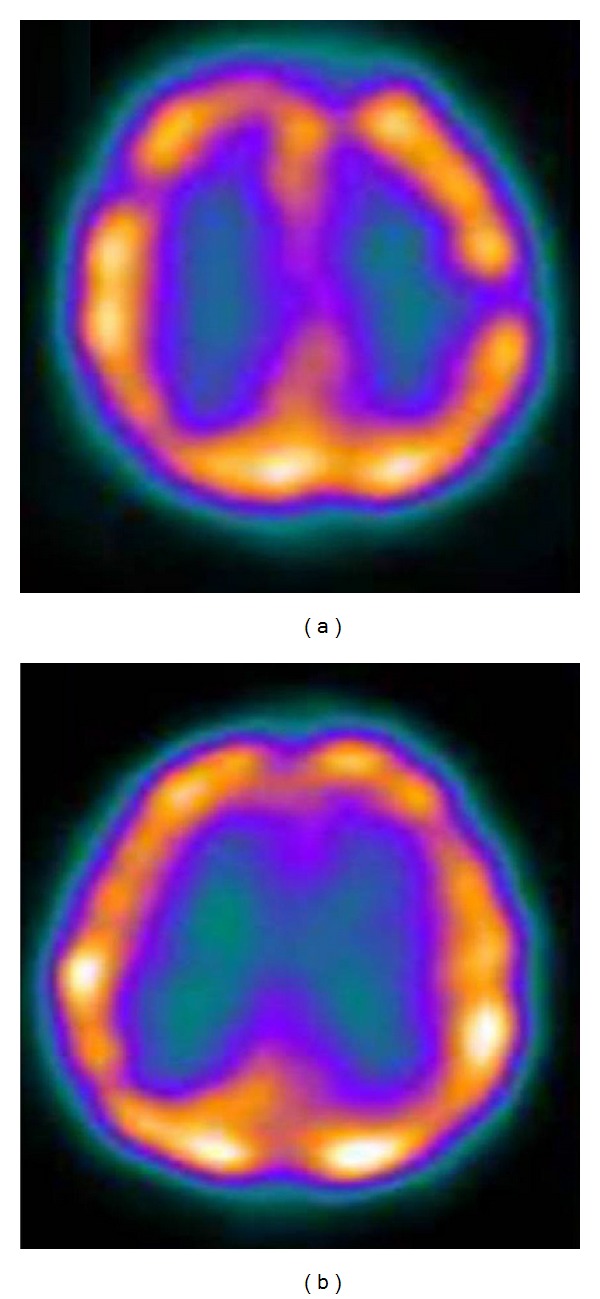
^99m^Tc-ECD brain SPECT transaxial image (a) preoperatively showing reduced radiotracer uptake involving left parietal lobe suggestive of hypoperfusion, (b) postoperatively showing increased radiotracer uptake involving left parietal lobe suggestive of improved perfusion.

**Figure 2:**
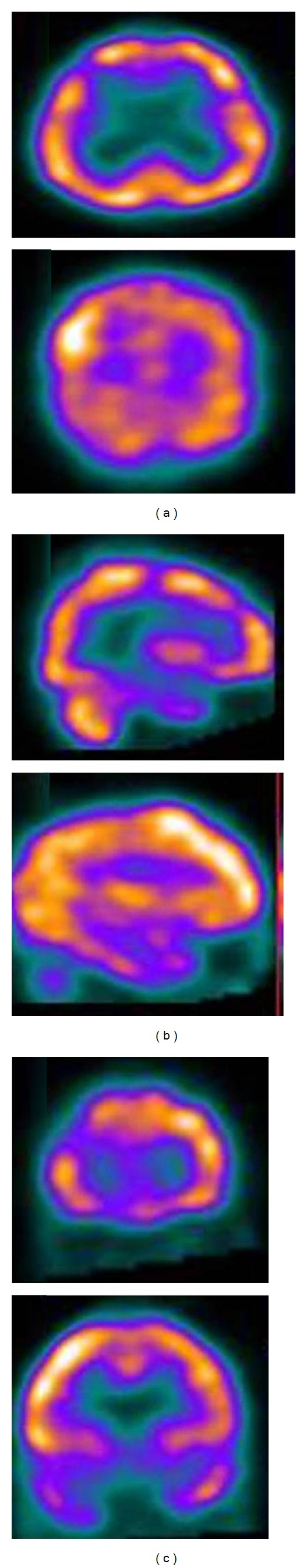
^99m^Tc-ECD brain SPECT preoperatively showing absent radiotracer uptake involving right frontal lobe suggestive of hypoperfusion, upper panel images axial (a), sagittal (b), and coronal (c), and postoperatively showing increased radiotracer uptake involving right frontal lobe suggestive of hyperperfusion, lower panel images axial (a), sagittal (b), and coronal (c).

**Figure 3:**
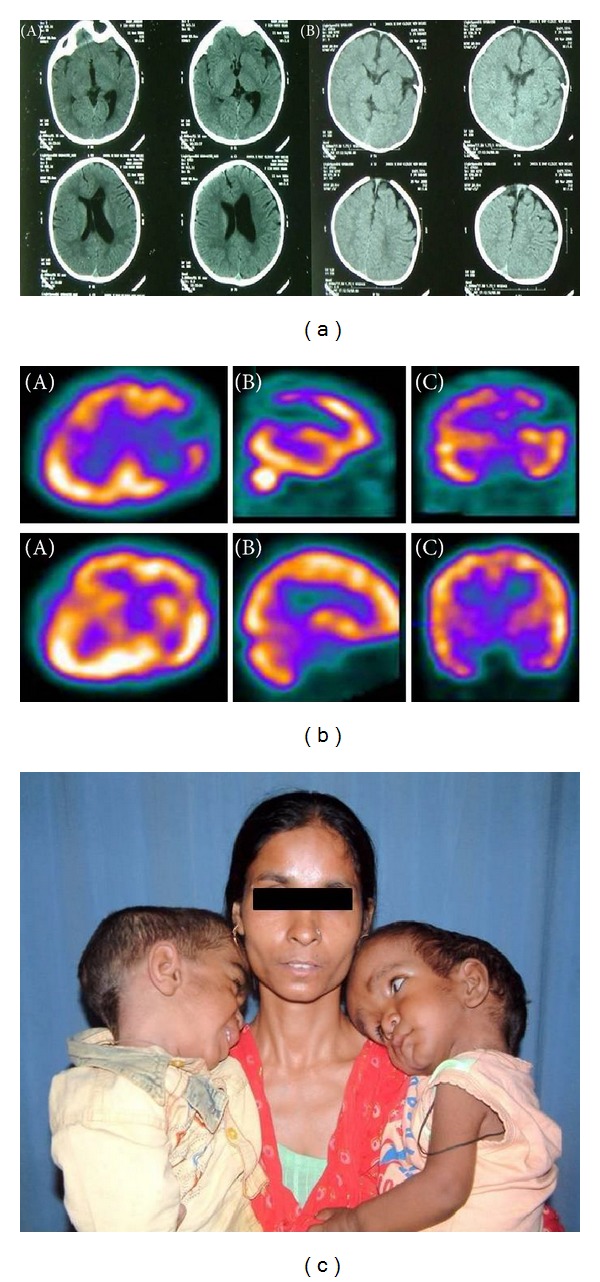
(a) Preoperative axial noncontrast CT (NCCT) skull images showing affected sulcal spaces of left frontoparietal lobe at the convexity and ventriculomegaly (A). Postoperative images showing decreased ventricular size and (B). (b) ^99m^Tc-ECD brain SPECT preoperatively showing absent radiotracer uptake involving left frontoparietal lobe suggestive of hypoperfusion, upper panel images axial (A), sagittal (B), and coronal (C), and postoperatively showing increased radiotracer uptake involving left frontoparietal lobe suggestive of hyperperfusion, lower panel images axial (A), sagittal (B), and coronal (C). (c) The preoperative clinical image of twin patients. Note the enlarged skull secondary to craniosynostoses and hydrocephalus in the patient on right arm of the mother.

**Figure 4:**
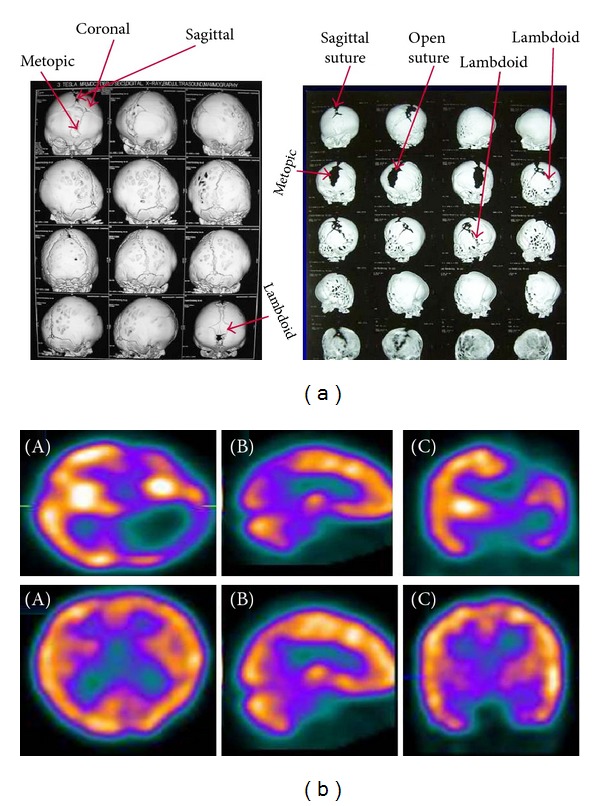
(a) CT volumetric rendering technique (VRT) images showing fusion of left fronto-parieto-temporo-occipital lobe sutures preoperatively (A) and release of all these sutures postoperatively (B). (b) ^99m^Tc-ECD brain SPECT preoperatively showing absent radiotracer uptake involving left fronto-parieto-temporo-occipital lobe suggestive of hypoperfusion, upper panel images axial (A), sagittal (B), and coronal (C), and postoperatively showing increased radiotracer uptake involving left fronto-parieto-temporo-occipital lobe suggestive of improved perfusion, lower panel images axial (A), sagittal (B), and coronal (C).

A total of 85 patient children were studied. There were 55 boys and 30 girls with age range being 1 to 38 months prior to corrective surgery. The observed detection rates of craniosynostosis were 72% (61/85), 94% (80/85), and 26% (4/15) by using X-ray, NCCT, and MRI, respectively. The lower detection rate with MRI is due to the fact that cortical bone is not well imaged by MRI due to low proton content; rather it detects any associated intracranial malformation/parenchymal abnormality, hydrocephalus, and raised intracranial tension. The images obtained in all children after injection of ^99m^Tc-ECD demonstrated three patterns of sutural (underlying cerebral cortical activity) activity: normal, absent, and increased. When correlated with surgical findings, “absent” indicated fused sutures and “increased” indicated fusing hyperactive sutures or sutures reacting to fusion elsewhere. So, a ^99m^Tc-ECD SPECT/CT hybrid imaging might prove to be superior over other imaging modalities as it can detect bony and underlying perfusion defects simultaneously. Most of the children showed evidence of hypoperfusion corresponding to the abnormally fused sutures on ^99m^Tc-ECD SPECT (Figures 1, 2, 3(b), and 4(b)), and few showed evidence of normal perfusion. For providing further information about the correlation of imaging modality, both pre- and postoperative noncontrast CT images of the skull of one case have been provided (Figure 3(a)). The corresponding ^99m^Tc-ECD SPECT scan image has been shown in Figure 3(b). The preoperative clinical photograph of twins with craniosynostoses have been provided (Figure 3(c)) with one of the twins having the radiological images as in Figures 3(a) and 3(b). For providing further information about the correlation of imaging modality, both pre- and postoperative CT volumetric rendering technique (VRT) images of the skull of one case have been provided (Figure 4(a)). The corresponding ^99m^Tc-ECD SPECT scan image has been shown in Figure 4(b). Postoperative follow-up imaging studies done at 3 months demonstrated a significant improvement of the perfusion defects and normalization of brain perfusion following surgical decompression (Table 2). Corresponding to this, the mean mental performance quotient (MPQ) increased significantly (*P* < 0.05) postoperatively only in those children (48/85) with absent perfusion defect preoperatively, when measured at 12 months during follow-up (Table 2).

## 4. Discussion

Craniosynostosis is one of the most common craniofacial anomalies with incidence of 1 in 2,500 live births. It can be “isolated” or “nonsyndromic” or may be “syndromic”. The former implies a sporadic occurring problem that usually affects a single suture causing a characteristic pattern of skull deformity, whereas the latter usually involves multiple sutures along with other associated malformations that include that of digital, skeletal, cardiac, or other organs. Though the syndromic variety can be diagnosed clinically, nonsyndromic variety requires imaging studies to confirm the diagnosis. Though plain X-ray of skull demonstrates a moderate to high sensitivity and specificity in diagnosing craniosynostosis, recent data support three-dimensional CT scan (3D-CT) as the best imaging modality with sensitivities ranging from 96 to 100% [11]. CT also detects associated intracranial pathology. In healthy children with head deformity including posterior plagiocephaly, X-ray skull is recommended, but syndromic craniosynostosis such as Apert, Crouzon, and Pfeiffer, nearly always requires CT imaging for further management including surgery. We compared different conventional imaging modalities and concluded the same. In our view, hybrid ^99m^Tc-ECD SPECT-CT imaging being a dual anatomical as well as functional imaging modality might prove superior in detecting both sutural and perfusion abnormalities and planning further management as well as prognosticating patients after surgery. This might also help surgeons to modify the surgical technique or change the timing of surgery, based on brain perfusion SPECT findings. However, in the present study, we could not carry out this hybrid imaging.

Though significant advancement has been made in understanding the pathogenesis of syndromic craniosynostosis, little is known about the pathogenesis of isolated or nonsyndromic variety. Pioneer work by Virchow showed that the primary defect lies within the suture that is later translated to the cranial base by an unknown mechanism [12]. Moss then postulates that the cranial base is the source of the primary defect, which translates its effects on the suture through the dura mater [13]. However, recently the focus is mainly on the dura which might be playing an integral role in determining the patency of the overlying suture. But, others believe that still there is evidence to suggest that the developing brain itself has a primary role in production of the craniosynostosis phenotype [14]. Whatever the etiological factors, there exists abnormality in perfusion and functional abnormalities in brain of the children with craniosynostosis [10, 15]. Previously, we have shown this by using ^99m^Tc-HMPAO SPECT [10].

In the present study, we used another isotope ^99m^Tc-ECD for SPECT imaging and found the preoperative studies in 56.5% (48/85) cases being abnormal in form of regional hypoperfusion in the underlying cerebral hemisphere, corresponding to the fused sutures (Figures 1–4). Every preoperative ^99m^Tc-ECD SPECT study was repeated at 3 months after surgery and demonstrated a significant postoperative improvement of the perfusion defects and normalization of brain perfusion following surgical decompression (Figures 1–4). Corresponding to this, the mean mental performance quotient (MPQ) significantly increased postoperatively only in those children (48/85) with absent perfusion defect preoperatively (*P* < 0.05), when measured at 1 year during follow-up. Also during follow-up at 1 year, the head circumference (measured by the cephalometric score) increased significantly (from mean of 59.4 in case of scaphocephaly and 77.1 in case of brachycephaly preoperatively to 65.2 in case of scaphocephaly and 83.6 in case of brachycephaly postoperatively) (*P* < 0.05). These findings strongly suggest that in craniosynostosis there is cerebral hypoperfusion at the microvascular tissue level, and the postoperative positive clinical and radiological impact can be attributed to the better function of the brain both by release of the stenosis and probably due to excellent collateral supply from the opposite side. Though the current indication of surgery for craniosynostosis is mainly for cosmetic reasons in most of the cases of an isolated craniosynostosis till now, present study shows, ^99m^Tc-ECD SPECT can prospectively identify patients benefiting from surgery.

## 5. Conclusions

Our study suggests that early surgery and release of craniosynostosis in patients with preoperative perfusion defects (absent on ^99m^Tc-ECD SPECT study) are beneficial as they lead to improved MPQ after surgery.

## Figures and Tables

**Table 1:** Clinical profile of children studied.

Variables	Number	Percentage
Age (months, mean ± SD)	15.6 ± 10.6	—
Male	55	64.7%
Syndromic type	15	17.6%
Nonsyndromic type		
Brachycephaly	36	51.4%
Scaphocephaly	18	25.7%
Trigonocephaly	10	14.3%
Plagiocephaly	04	5.7%
Oxycephaly	02	2.8
Sutures involved		
Coronal	36	42.4%
Sagittal	18	21.2%
Metopic	10	11.7%
Lambdoid	02	2.3%
Multiple	19	22.3%
Fundoscopy (papilledema)	44	51.7%
Hydrocephalus or raised intracranial pressure	21	24.7%
Microcephaly	16	18.8%
Asymptomatic	59	69.4%

**Table 2:** Results of preoperative and postoperative investigations.

Conventional imaging findings (number of patients)	Preoperative ^99m^Tc-ECD SPECT	Dural vascularity at surgery	Mean (±SD) mental performance quotients (MPQ)		Postoperative ^99m^Tc-ECD SPECT
			Pre-op	Post-op	
Fusion of bilateral coronal, lambdoid, and sagittal sutures (*N* = 19)	Absent	Scanty	85.1 (17.9)	92.6 (16.6)*	Normal
Fusion of left coronal and sagittal suture (*N* = 18)	Absent	Scanty	86.2 (15.2)	94.2 (13.4)*	Normal
Fusion of bilateral coronal sutures (*N* = 11)	Absent	Scanty	87.2 (14.1)	93.8 (15.7)*	Normal to increased
Fusion of bilateral coronal sutures (*N* = 07)	Normal	Scanty	89.8 (14.6)	93.1 (14.8)	Normal to increased
Fusion of left coronal suture (*N* = 04)	Normal	Normal	92.7 (15.3)	94.5 (13.7)	Normal
Fusion of right coronal suture (*N* = 14)	Normal	Normal	93.6 (14.9)	95.1 (13.6)	Normal
Fusion of metopic suture (*N* = 10)	Normal	Scanty	92.5 (12.7)	94.3 (16.4)	Normal
Fusion of right lambdoid suture (*N* = 02)	Normal	Normal	94.9 (13.1)	95.3 (15.5)	Normal

*P value < 0.05.

## References

[1] Panigrahi I (2011). Craniosynostosis genetics: the mystery unfolds. *Indian Journal of Human Genetics*.

[2] Barik M, Bajpai M, Das RR, Panda SS (2013). Study of environmental and genetic factors in children with craniosynostosis: a case-control study. *Journal of Pediatric Neurosciences*.

[3] Coussens AK, Wilkinson CR, Hughes IP (2007). Unravelling the molecular control of calvarial suture fusion in children with craniosynostosis. *BMC Genomics*.

[4] Lee HQ, Hutson JM, Wray AC (2012). Changing epidemiology of nonsyndromic craniosynostosis and revisiting the risk factors. *The Journal of Craniofacial Surgery*.

[5] Nagaraja S, Anslow P, Winter B (2013). Craniosynostosis. *Clinical Radiology*.

[6] Iivanainen M, Launes J, Pihko H, Nikkinen P, Lindroth L (1990). Single-photon emission computed tomography of brain perfusion: analysis of 60 paediatric cases. *Developmental Medicine and Child Neurology*.

[7] Lekovic GP, Bristol RE, Rekate HL (2004). Cognitive impact of craniosynostosis. *Seminars in Pediatric Neurology*.

[8] Clijmans T, Mommaerts M, Gelaude F (2008). Skull reconstruction planning transfer to the operation room by thin metallic templates: clinical results. *Journal of Cranio-Maxillofacial Surgery*.

[9] Brinkmann BH, Jones DT, Stead M (2012). Statistical parametric mapping demonstrates asymmetric uptake with Tc-99m ECD and Tc-99m HMPAO SPECT in normal brain. *Journal of Cerebral Blood Flow and Metabolism*.

[10] Sen A, Dougal P, Padhy AK (1995). Technetium-99m-HMPAO SPECT cerebral blood flow study in children with craniosynostosis. *Journal of Nuclear Medicine*.

[11] Medina LS, Richardson RR, Crone K (2002). Children with suspected craniosynostosis: a cost-effectiveness analysis of diagnostic strategies. *American Journal of Roentgenology*.

[12] Foley LM, Fellows-Mayle W, Hitchens TK (2009). Age-related peridural hyperemia in craniosynostotic rabbits. *Child’s Nervous System*.

[13] Cohen MM (2005). Editorial: perspectives on craniosynostosis. *American Journal of Medical Genetics*.

[14] Aldridge K, Marsh JL, Govier D, Richtsmeier JT (2002). Central nervous system phenotypes in craniosynostosis. *Journal of Anatomy*.

[15] Schaller BJ, Filis A, Merten HA, Buchfelder M (2012). Premature craniosynostosis—the role of skull base surgery in its correction. A surgical and radiological experience of 172 operated infants/children. *Journal of Cranio-Maxillofacial Surgery*.

